# Parental care and sibling competition independently increase phenotypic variation among burying beetle siblings

**DOI:** 10.1111/evo.13607

**Published:** 2018-10-04

**Authors:** Matthew Schrader, Benjamin J. M. Jarrett, Rebecca M. Kilner

**Affiliations:** ^1^ Department of Biology University of the South Sewanee Tennessee 37383; ^2^ Department of Zoology University of Cambridge Cambridge CB2 3EJ United Kingdom; ^3^ Department of Entomology Michigan State University East Lansing Michigan 48824

**Keywords:** Body size evolution, *Nicrophorus vespilloides*, parental care, variation

## Abstract

Several recent hypotheses suggest that parental care can influence the extent of phenotypic variation within populations; however, there have been few tests of these ideas. We exploited the facultative nature of posthatching parental care in the burying beetle, *Nicrophorus vespilloides*, to test whether parental care influences the expression of phenotypic variation in an important fitness trait (body size). We found that parental care and brood size (which influences sibling competition) had positive and independent effects on variation in body size. First, the mean coefficient of variation (CV) of body size was significantly greater in broods that received care than in those that did not. Second, CV body size increased with brood size in both parental care treatments. These results are not consistent with predictions from recent hypotheses that predict parental care will reduce phenotypic variation among siblings. The positive effects of parental care and brood size on phenotypic variation that we observed are likely due to sibling competition for access to provisioning parents and competition for limiting resources contained in the breeding carcass. Our results suggest that future theory linking parental care to the generation and maintenance of phenotypic variation must integrate the nature of interactions among family members.

In animals with parental care, the amount, or quality of care that young receive can have an important impact on their phenotypic development and fitness (Smiseth et al. 2012). There is now a vast literature examining how parental care influences the mean value of traits (e.g., size) expressed in offspring (Roff 1992; Eggert et al. 1998; Hunt and Simmons 2000). However, with the exception of hatching asynchrony and its consequences (Smiseth et al. 2007b), less attention has been paid to how interactions within the family influence phenotypic variation among dependent offspring within the same brood. This is somewhat surprising, as there are several mechanisms by which parental care, and sibling interactions, can influence the amount of phenotypic (and in some cases genetic) variation that is maintained within a population (e.g., Snell‐Rood et al. 2016; Wade 1998; Wolf and Brodie 1998).

Snell‐Rood et al. (2016) recently reviewed three hypotheses linking parental care to the maintenance of phenotypic and genetic variation (the environmental stress, compensation, and relaxed selection hypotheses). These three hypotheses each predict that parental care will reduce the amount of phenotypic variation expressed within a group of siblings; however, they focus on different mechanisms linking parental care to the maintenance of phenotypic variation. The environmental stress hypothesis proposes that the phenotypic effect of a mutation depends upon the environmental context in which it is expressed and that mutations generally have a greater impact on the phenotype in stressful environments than in benign environments (Kondrashov and Houle 1994; Martin and Lenormand 2006). Parental care often functions to buffer offspring from environmental stress (Royle et al. 2012; Pilakouta et al. 2015) and may thus dampen the phenotypic effect of mutations, thereby hiding these mutations from natural selection. This hypothesis makes two predictions. First, it predicts that parental care will reduce phenotypic variation among siblings. This effect of parental care on phenotypic variation could be detected experimentally by comparing phenotypic variation among groups that receive care and those that do not. Second, this environmental stress hypothesis predicts that the buffering effect of parental care will relax selection on offspring phenotype and lead to a build up of genetic variation. Thus, populations that experience parental care may harbor more standing genetic variation than populations maintained without parental care.

The compensation hypothesis also proposes that parental care influences the phenotypic effect of mutations. However, it suggests that parental care is a means by which parents can directly compensate for the negative effect of a deleterious mutation on offspring phenotype (e.g., Pilakouta et al. 2015; Mattey et al. 2018). This hypothesis therefore also predicts that parental care can reduce phenotypic variation among siblings. It is most applicable to species with direct and extended parental care, in which parents can potentially detect and compensate for deleterious effects of a new mutation expressed in their offspring. For example, parents may compensate for a mutation that causes offspring to exhibit low growth by increasing the rate at which they provision young (Lock et al. 2007; Mattey et al. 2018).

The relaxed selection hypothesis is based upon theory predicting that the intensity of selection will be stronger on alleles that impact fitness in frequently occupied environments than on alleles that impact fitness in rarely occupied environments (Kawecki 1994; Whitlock 1996; Van Dyken and Wade 2010; Snell‐Rood et al. 2010). For example, in species in which young typically develop with high levels of parental care (i.e., a high‐care environment), mutations that impact fitness only in the absence of parental care (i.e., in a low‐care environment) will be under weak selection. This will lead to an accumulation of cryptic genetic variation for traits that impact fitness in the low‐care environment. In principle, this cryptic genetic variation can be exposed by rearing individuals adapted to a high‐care environment in a low‐care environment (Snell‐Rood et al. 2016).

In addition to the three hypotheses reviewed by Snell‐Rood et al. (2016), Wade (1998) has suggested that parental effects (including parental care) can alter the level of selection on offspring and that this will affect the amount of genetic variation that is maintained within a population. According to this hypothesis, which we refer to as the parental effects hypothesis, parental care can cause offspring with different genotypes to have the same phenotype because they occupy the same parentally provided environment. Under these circumstances, selection on offspring is likely to operate at the level of entire families, because brood‐mates share the same parentally induced phenotype (Wade 1998; Wolf 2000). This will reduce phenotypic variation among siblings but result in more genetic variance being maintained at equilibrium (Wade 1998). Importantly, the hypotheses described above are not mutually exclusive (Snell‐Rood et al. 2016). For example, parental care might simultaneously reduce the phenotypic effect of a mutation expressed in offspring and alter the strength or level of selection that acts on that mutation.

The hypotheses described in the preceding paragraphs describe ways in which parental care might influence phenotypic (and possibly genetic) variation within families without explicitly considering the impact that dependent siblings may have upon one another through competition (or cooperation) for parentally supplied resources. However, there is compelling evidence that sibling interactions may also have an important impact on the development of offspring phenotypes in some species (Mock and Parker 1997; Roulin and Dreiss 2012; Rebar et al. unpubl. ms). For example, the burying beetle (*Nicrophorus vespilloides*) exhibits complex pre‐ and posthatching parental care and also displays hatching asynchrony (Smiseth et al. 2007b; Royle et al. 2013). As a result, early and late hatching larvae differ in their access to posthatching parental care (Smiseth et al. 2007b). This type of asymmetric competition for access to parental care has the potential to increase within‐brood variation in offspring phenotypes, such as body size, that are influenced by access to parental care.

These hypotheses linking interactions within the family to the maintenance of phenotypic variation have important implications for adaptation and evolutionary diversification. For example, if parental care reduces the amount of phenotypic variation that is expressed, parental care can lead to a build up of genetic variation within a population, possibly fueling adaptation to novel environments (Snell‐Rood et al. 2016). On the other hand, parental care may reduce the potential for populations to respond to selection. For example, parental care may increase phenotypic variation among siblings due to competition for access to care. If such an increase in phenotypic variation is due mainly to an increase in environmental variation, then the narrow‐sense heritability and the potential for a trait to respond to selection will both be reduced. In addition, parental care can be a source of indirect genetic effects and direct‐indirect genetic covariances, which can combine to reduce total heritability and limit the response to selection in the short term (e.g., Rauter and Moore 2002; Head et al. 2012). Despite the potential importance of parental care in influencing phenotypic variation and adaptation, few empirical studies have directly examined whether and how the presence of parental care influences the level of phenotypic variation that is expressed in a given trait within a brood. In addition, studies that have attempted to estimate the impact of parental care on the maintenance of phenotypic variation have focused on species in which care is indirect and young develop individually (e.g., *Onthophagus* beetles; Snell‐Rood et al. 2016). Whether parental care influences the expression of phenotypic variation in species with direct care and sibling rivalry has been relatively unexplored. Here, we attempt to fill this gap by examining the links between posthatching parental care, sibling competition, and the expression of phenotypic variation in body size in the burying beetle, *N. vespilloides*, a species in which parental care can be manipulated experimentally and dependent offspring compete for parental care (Smiseth et al. 2007b).

## Methods

Our experiment focused on the burying beetle, *N. vespilloides*. This species breeds on vertebrate carrion and displays pre‐ and posthatching parental care, which is typical of most burying beetle species (reviewed in Scott 1998; Royle et al. 2013). Prehatching care lasts approximately 3 days and involves shaving the carcass, rolling it into a ball and burying it, while smearing the surface of the carcass with anti‐microbial exudates. Posthatching care involves feeding begging larvae fluids, maintaining the carcass, and defending the carcass and brood from competitors. The duration of posthatching care is highly variable, but it has most effect on offspring fitness in the first 24 hours after the larvae hatch and arrive at the carcass (Eggert et al. 1998). Several studies have shown that posthatching parental care in *N. vespilloides* increases larval survival and average body size (Eggert et al. 1998; Schrader et al. 2015b). However, posthatching care is not necessary for larval survival and populations rapidly adapt to its removal (Schrader et al. 2015a; Schrader et al. 2017; Jarrett et al. 2018). The facultative nature of posthatching parental care makes this an excellent species in which to examine the effect of parental care on the expression and maintenance of phenotypic variation (Smiseth et al. 2007a; Snell‐Rood et al. 2016; Jarrett et al. 2017).

The beetles used in this experiment were part of a laboratory population housed at the University of Cambridge, and constituted the first generation of a large‐scale selection experiment reported in Jarrett et al. (2017). The data we focus on here were collected before any artificial selection was imposed, and the analyses we present here have not been published before. This population was founded in 2013 from adults collected under license from two locations in Cambridgeshire, UK (Byron's Pool and Wicken Fen). Adults were housed individually in plastic boxes (12 × 8 × 2 cm) containing moist soil (Miracle Grow) and were fed approximately 0.3 g of minced beef twice per week. All of the adults in this experiment were bred 17 days after they eclosed. Each breeding pair was created by randomly pairing an unrelated male and female. Pairs were then assigned to one of two parental care treatments: Full Care and No Care. Each breeding pair was placed into a larger breeding box (17 × 12 × 6 cm) half‐filled with moist soil and containing a freshly thawed and weighed mouse carcass. Carcass mass varied between 6.6 and 19.7 g (mean ± SD = 12.24 ± 2.5 g).

In the Full Care treatment, we left both parents in the breeding box with the larvae until the larvae began to disperse away from the carcass to pupate (∼8 days after pairing). Although parents in this treatment were not forced to provide care to their larvae, previous work has shown that under these conditions the parents care for the brood for at least the first 24 hours after hatching (Jarrett et al. 2018), which is the time period during which parental care is most beneficial to larvae (Eggert et al. 1998). In the No Care treatment, we removed both parents from the breeding box 53 hours after pairing (as in Schrader et al. 2015b; Schrader et al. 2017; Jarrett et al. 2017). By this time parents have finished preparing the carcass and completed egg laying; however, the eggs have not yet hatched (Boncoraglio and Kilner 2012; Schrader et al. 2015b). Eight days after pairing, and when two or more larvae were starting to disperse away from the carcass, we counted the number of dispersing larvae in all broods and weighed the entire mass of the brood. We then placed each larva in an individual 2 × 2 × 2 cm cell within a 25‐cell plastic eclosion box (10 × 10 × 2 cm) and covered the entire brood with a layer of damp peat. We used a different eclosion box for each brood and each eclosion box was covered with a plastic lid. Upon eclosion (17 days after dispersal), we took a digital photograph of each adult beetle. We used the resulting images to measure adult pronotum width (hereafter “body size”). The protocol we used to measure body size from these images is described in Jarrett et al. (2017).

We tested whether the presence of posthatching parental care influences phenotypic variation in body size, by measuring variation in adult body size (pronotum width in mm) within *N. vespilloides* families that were reared with or without posthatching parental care (Full Care and No Care treatments respectively). We focus specifically on body size because it is very likely to be linked to fitness in burying beetles (e.g., Bartlett and Ashworth 1988; Otronen 1988; Steiger 2013; Pascoal et al. 2018). For each brood produced within the two parental care treatments (167 Full Care broods and 91 No Care broods), we calculated the mean, standard deviation, and coefficient of variation of body size when offspring matured into adults. The sexes were combined in these calculations because there is no sexual dimorphism in body size in this species (Jarrett et al. 2017). Because there was a significant difference between the two treatments in mean body size (see Results below), we focused further analyses on the coefficient of variation of body size (CV _body size_) within each brood. This measurement standardizes the variance by the mean and is often used to compare standard deviations between populations with different means (Sokal and Rohlf 1995).

Removing posthatching parental care reduces brood size in this species (Eggert et al. 1998). So, to ensure that there was broad overlap in brood size (and the potential for sibling competition) between the No Care and Full Care treatments we restricted our analysis to broods with >4 dispersing larvae. This eliminated 21 No Care broods and three Full Care broods, but resulted in overlap in the brood size distributions of the two treatment groups (see Results below). Preliminary analyses indicated that CV _body size_ increased with brood size (correlation between brood size and CV _body size_: *r* = 0.32, *P* = 1.7 × 10 ^−7^, *n* = 258), but was not significantly correlated with carcass mass (correlation between carcass mass and CV _body size_: *r* = –0.10 *P* = 0.09, *n* = 258). We therefore tested whether the Full Care and No Care treatments differed in their average CV _body size_ using an ANCOVA with brood size as a covariate. We initially included the treatment by brood size interaction in the model to test the homogeneity of slopes assumption. This interaction was not significant (ANCOVA, treatment by brood size interaction: *F*_1,255_ *=* 0.13, *P* = 0.72) so it was removed from the final model.

The coefficient of variation is the ratio of the standard deviation to the mean. Thus, the increase in CV _body size_ with brood size may just reflect a decrease in mean body size with increasing brood size (which would cause this ratio to increase). Several studies have shown that mean larval mass decreases with brood size in *N. vespilloides* (e.g., Smiseth et al. 2014; Schrader et al. 2015a) and we observed a similar pattern in this experiment (correlation between brood size and mean larval mass: *r* = –0.48, *P* = 2.2 × 10 ^−16^, *n* = 258). Thus, to confirm that the increase in CV _body size_ was not driven by a decline in mean larval mass, we repeated the analyses described above using the standard deviation of adult body size (SD _body size_) as the response variable instead of the CV _body size_. The results of this analysis are the same as the analysis of CV _body size_, suggesting that our results were not driven by differences in means between treatments. For this reason, we focus only on the results of the analysis of CV _body size_. All statistical analyses were conducted in R version 3.3.1 (R Core Team 2016).

## Results

We measured the size of 4332 adult beetles from 258 families (167 Full Care broods and 91 No Care broods). Adults that received posthatching parental care as larvae were slightly but significantly larger than those that received no posthatching parental care (Full Care mean pronotum width = 4.66 mm, No Care mean pronotum width = 4.55 mm; *t*‐test comparing family means between the Full Care and No Care treatments: *t*_204.37_ = 2.84, *P* = 0.0049). This result is consistent with previous studies showing that parental care has a positive impact on larval growth and mass at dispersal, which determines adult body size (Eggert et al. 1998; Smiseth et al. 2007a; Schrader et al. 2015b).

**Figure 1:**
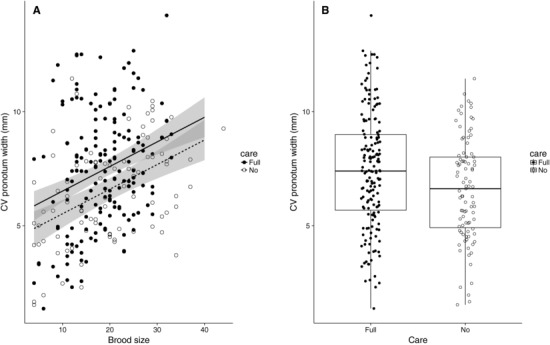
(A) The relationship between the coefficient of variation (CV) of adult body size (pronotum width in mm) and brood size when beetles received Full Care (black symbols, solid line) or No Care (open symbols, dashed line) as larvae. Each data point represents a single brood. Lines and 95% confidence intervals are from the ANCOVA described in the text. (B) The distribution of coefficients of variation in the Full Care and No Care treatments, illustrating the overall difference between the two treatments in the mean CV of pronotum width.

Variation in adult body size within broods (measured as CV _body size_) increased with brood size (ANCOVA, effect of brood size: *F*_1,256_ = 30.02, *P* = 1.03 × 10^−7^) and was significantly higher in the Full Care treatment than the No Care treatment (ANCOVA, effect of care: *F*_1, 256_ = 10.85, *P* = 0.0011; Full Care, mean *CV*_body size_ = 7.4%; No Care, mean *CV*_body size_ = 6.6%; Fig. 1).

## Discussion

Most of the theory linking parental care (and the social interactions that accompany it) to phenotypic/genetic variation predicts that the presence of parental care will reduce phenotypic variation among siblings (Table 1). Preliminary tests of two of these hypotheses (the environmental stress and relaxed selection hypotheses) using dung beetles (*Onthophagus spp*) have provided some support for this prediction. For example, Snell‐Rood et al. (2016) found that the phenotypic impact of novel mutations induced by radiation were greater in *O. gazella* that were exposed to conditions simulating low levels of parental care than those exposed to conditions simulating high levels of parental care. In addition, comparisons between two *O. taurus* populations that differ in parental care behaviors revealed that the high care population exhibited greater phenotypic variation in body size when reared under conditions simulating low care than conditions simulating high care (Snell‐Rood et al. 2016).

**Table 1:** A comparison of the predictions of hypotheses linking interactions within the family to the maintenance of phenotypic variation

Hypothesis	Treatment expected to exhibit the greatest phenotypic variation	References
Environmental stress	No care	Snell‐Rood et al. 2016
Compensation	No care	Snell‐Rood et al. 2016
Relaxed selection	No care	Snell‐Rood et al. 2016
Parental effects	No care	Wade 1998
Sibling competition	Full care	Smiseth et al. 2007b

For each hypothesis, we list the environment (Full Care or No Care) in which phenotypic variation is expected to be greatest.

In contrast to these experiments, we found that phenotypic variation in adult body size was significantly greater in *N. vespilloides* broods that had been reared with posthatching parental care than in broods that had been reared without (Fig. 1). This pattern is opposite to that predicted by four of the major hypotheses that have proposed a link between parental care and the expression of phenotypic/genetic variation (Table 1), but is consistent with the hypothesis that sibling‐competition for access to parental care increases phenotypic variation in body size among siblings. Studies of other organisms (e.g., fish in aquaculture) often use the coefficient of variation of body size within a population as an index of the competitive environment that individuals experience during growth (Jobling 1995; Majorovich et al. 2018), with a higher coefficient of variation indicating greater competition. The importance of sibling competition for parental care in generating phenotypic variation in body size is also supported by previous studies of *N. vespilloides*. For example, Smiseth et al. (2007b) examined the effect of parental care on the growth trajectories of asynchronously hatched, *N. vespilloides* larvae and found that earlier hatched (senior) larvae grew at a higher rate than later hatched (junior) larvae when parents were allowed to care for offspring. However, when larvae were deprived of posthatching parental care, junior, and senior larvae grew at the same rate. The untested implication of that previous study is that there should be more variance in larval mass at dispersal (and hence adult body size) when larvae receive care than when they do not. Our result suggests that the growth trajectories described by Smiseth et al. (2007a) generate greater phenotypic variance in adult body size within broods that received parental care than within those that did not.

Although posthatching parental care had a significant impact on variation in body size, brood size had a much larger positive impact on variation in adult body size (parental care partial η^2^ = 0.041; brood size partial η^2^ = 0.11). This result may also be driven by competition among siblings for a parentally supplied resource. Previous studies of *N. vespilloides* have shown that mean larval mass at dispersal (which determines adult body size) declines with increasing brood size (e.g., Smiseth et al. 2014; Schrader et al. 2015a) and we observed the same pattern here. This decline is likely a consequence of increasing sibling competition for a fixed amount of energy contained in the carcass. It is possible that the increase in CV _body size_ with increasing brood size is also a manifestation of sibling competition for a fixed pool of resources and that this competition generates variation in body size within a brood even in the absence of posthatching parental care.

An alternative explanation for our results is that posthatching parental care weakens selection on body size, and as a consequence more phenotypic variation in adult size is maintained with care than without (because selective mortality is reduced). Testing this hypothesis requires estimates of the strength or form of selection on larval traits with and without parental care. Although we currently lack such estimates, recent studies have shown that adaptation to the removal of posthatching parental care in *N. vespilloides* populations involves rapid increases in both larval survival and mean larval mass at dispersal (Schrader et al. 2015b; Schrader et al. 2017). Thus, strong selection associated with the removal of parental care may reduce phenotypic variation in adult body size in the No Care treatment compared to the Full Care treatment. Testing this hypothesis will require following individually marked larvae through development, something that has to our knowledge not been done. Although relaxed selection may explain the mean difference in CV _body size_ between the Full Care and No Care populations, the increase in CV _body size_ with brood size is unlikely to be explained by relaxed selection. In fact, the increased level of competition between siblings in large broods suggests that selection may actually be stronger in large broods than in small broods. If this were the case, then we would expect to see a decrease in phenotypic variation in body size with increasing brood size (due to selective losses). This is the opposite of what we observed.

Finally, our experiment revealed extensive variability among families in CV _body size_, even within care treatments and brood sizes. For example, at a brood size of 20 there was a twofold difference between the largest and smallest measures of CV _body size_ (see Fig. 1A). Understanding what generates this variation remains unknown. It may be that variation in body size within a family is due in part to heritable aspects of the social environment experienced by siblings, as a type of indirect genetic effect (Moore et al. 1997; Wolf et al. 1998; Marjanovic et al. 2018). If this is the case, the variation in body size within a family can be treated as a quantitative trait in itself (i.e., it is a form of “inherited variability”; see Marjanovic et al. 2018). Quantitative genetic studies of burying beetles have been used to study the indirect genetic effects that arise due to parental care and their impact on mean trait values (e.g., Rauter and Moore 2002; Head et al. 2012). Much less is known about whether indirect genetic effects contribute to variability in trait values in burying beetles (or other organisms for that matter). Dissecting the causes and consequences of variability within families is an exciting avenue for future studies for several reasons. For example, variation in body size within families reflects the degree to which this trait is canalized: the lower the variation, the greater the extent of canalization. Recent theoretical work indicates that canalization may have a social genetic component that can respond to selection (Marjanovic et al. 2018). Our study identifies two social conditions in *N. vespilloides* (parental care and brood size) that influence the degree to which body size is canalized. Thus, selection on one or both of these social conditions may lead to the evolution of canalization (or decanalization) in body size (Marjanovic et al. 2018). Experiments by Jarrett et al. (2017) examined the impact of the social environment on the evolution of average body size in experimental *N. vespilloides* populations. It may be possible to use a similar approach to study canalization or decanalization in body size by exerting family level selection on CV _body size_.

In conclusion, our results suggest that interactions between family members (i.e., interactions between parents and offspring and among dependent siblings within the same brood) have significant and largely independent impacts on the amount of phenotypic variation in body size that is expressed within a family. Our results are not consistent with the predictions of many of the hypotheses that have been proposed linking parental care and phenotypic variation, suggesting that the suitability of these hypotheses to specific systems may depend critically upon the type of care provided by parents (whether it is direct or indirect) and the nature of interactions among siblings (whether they are competitive or cooperative). Specifically, our results suggest that in *N. vespilloides*, sibling rivalry plays an important role in generating variation in adult body size. When parents are present, competition among brood mates for access to parental care may generate phenotypic variation in larval mass (and thus, adult body size) among siblings within the same brood. Importantly, it appears that competition between larvae during self‐feeding (i.e., in the absence of parental provisioning) also generates phenotypic variation in body size. Future studies examining the relationship between parental care and the maintenance of phenotypic variation should explicitly incorporate interactions between brood mates as these interactions may be as important as parental care in generating phenotypic variation. Finally, our results suggest that burying beetles may be an excellent system in which to study the impact of the social environment on inherited variability and its evolutionary consequences.

Associate Editor: J. McGlothlin

Handling Editor: Mohamed A. F. Noor
